# A cross-sectional study of the association between persistent organochlorine pollutants and diabetes

**DOI:** 10.1186/1476-069X-4-28

**Published:** 2005-11-29

**Authors:** Lars Rylander, Anna Rignell-Hydbom, Lars Hagmar

**Affiliations:** 1 Division of Occupational and Environmental Medicine and Psychiatric Epidemiology, Department of Laboratory Medicine, University Hospital, SE-221 85 Lund, Sweden

## Abstract

**Background:**

Experimental evidence supports the hypothesis that persistent organochlorine pollutants (POPs) may cause type 2 diabetes mellitus, whereas there is no fully convincing epidemiological evidence for such an association. In Sweden the most important source of POP exposure is fatty fish. We have assessed the association between serum levels of POPs and prevalence of diabetes in Swedish fishermen and their wives, with high consumption of fatty fish from the Baltic Sea.

**Methods:**

In 196 men (median age 60 years) and 184 women (median age 64 years), we analyzed 2,2',4,4',5,5'-hexachlorobiphenyl (CB-153) and 1,1-dichloro-2,2-bis(p-chlorophenyl)-ethylene (p,p'-DDE) in serum using gas chromatography-mass spectrometry. The participants were asked if they had diabetes and, if so, since which year and about medication and diet. The Odds Ratios (OR) for diabetes with respect to continuous exposure variables were analyzed with logistic regression, adjusting for potential confounders. Moreover trends of diabetes prevalence with respect to trichotomized exposure variables were tested with Jonckheere-Terpstra's test.

**Results:**

Six percent of the men and 5% of the women had diabetes. After confounder adjustment CB-153 was significantly associated with diabetes prevalence using both categorized and continuous exposure data (an increase of 100 ng/g lipid corresponded to an OR of 1.16, 95% confidence interval [CI] 1.03, 1.32, p = 0.03). Similar associations were observed for p,p'-DDE (an increase of 100 ng/g lipid corresponded to an OR of 1.05, 95% CI 1.01, 1.09, p = 0.006). Gender stratified analyses showed among men consistent positive associations with CB-153, but a more ambiguous pattern with respect to DDE. In contrast, among the women the associations with p,p'-DDE were stronger than with CB-153.

**Conclusion:**

The study provides support that POP exposure might contribute to type 2 diabetes mellitus.

## Introduction

It is widely believed that the increase in incidence of type 2 diabetes mellitus (T2DM) and obesity is the result of a complex interplay between genetic and environmental factors [1]. T2DM is due to resistance to insulin action and a relative deficiency of insulin. Age, obesity, central adiposity, lack of physical activity, dietary glycemic load, as well as certain genotypic variants are the main factors identified as responsible for the disease [2,3].

Exposure to dioxin has been linked to drastic reductions in glucose uptake in guinea pigs, mice and rats, *in vivo *as well as *in vitro *[4], leading to speculation that chronic low-level exposure to dioxins might be a risk factor for T2DM [5].

Previous epidemiological studies have recently been reviewed [6]. Several studies have linked high dioxin burdens to increased risks of T2DM or modified glucose metabolism [7-10]. A Belgian study published later showed highly significant elevation of serum levels of dioxins and polychlorinated biphenyls (PCBs) among patients with type T2DM [11]. Moreover, also chlorinated insecticides have been associated with T2DM. In a group of pesticide users and an unexposed group, Morgan et al [12] found that subjects with T2DM had higher blood levels of dichlorodiphenyl trichloroethane (DDT) and 1,1-dichloro-2,2-bis(p-chlorophenyl)-ethylene (p,p'-DDE), which is the major metabolite of DDT.

A major difficulty in these studies is, however, that exposure occurred sometimes many years before the epidemiologic study, which makes it difficult to determine whether the higher serum levels of persistent organochlorine pollutants (POPs) in diabetics truly reflect a higher exposure to these pollutants, which in turn may contribute to diabetogenesis, or whether they are merely the consequence of T2DM-induced metabolic perturbations facilitating the accumulation of these pollutants. Thus, the possibility of a reversed causality cannot be excluded.

In Sweden, consumption of fatty fish from the Baltic Sea, off the Swedish east coast, is the single major exposure source for POPs, and cohorts of professional fishermen and their families from the Swedish east coast have been found to constitute excellent study bases for epidemiological evaluations of human health effects of POPs [13,14]. We have chosen to use 2,2',4,4',5,5'-hexachlorobiphenyl (CB-153) as a biomarker for POP exposure, because it correlates very well (r ≥ 0.98) with both total PCB concentration in plasma and serum from Swedish subjects [15,16] and with the 2,3,7,8-tetrachlorodibenzo-*p*-dioxin (TCDD) toxic equivalent (TEQ) in plasma from PCB (r = 0.89) as well as the total POP derived TEQ (r = 0.74) in plasma in American Vietnam veterans [17]. Another relevant exposure biomarker is the anti-androgenic compound p,p'-DDE.

The aim of the present study was to assess the associations between biomarkers for POP and prevalence of diabetes in high exposure cohorts of middle-aged and elderly men and women. The results showed an association between the POP markers in serum and prevalence of diabetes.

## Methods

### Study population and interview

Previously established cohorts of professional fishermen and their wives from the Swedish east coast [14,18] were linked to the Swedish Population Register. A postal questionnaire was sent in year 2000 to 1500 fishermen and 1291 fishermen's wives that were born between 1920 and 1954, living in Sweden and still alive at 31 December 1999 [28]. There were 813 men (54%) and 779 women (77%) who responded to the questionnaire. Out of them 510 men and 596 women were positive to participate in future clinical studies. We invited a subset of them to a study mainly focused on bone mineral density [11], but also comprising other potential health effects of POP exposure. The aim was to include 200 men and 200 women in the study, and we consecutively contacted subjects by phone for agreements until enough subjects were recruited. Details of the recruitment process have been given elsewhere [19]. The final study groups comprised 196 men and 184 women.

The participants were interviewed, using a standardized questionnaire. The subjects were asked if they had diabetes and, if so, since which year. Moreover, they were asked if they had per oral antidiabetic drugs, insulin or were on diet. We measured current weight and height. In addition, they were asked about their weight at the age of 25 years. Descriptive data for the participants are shown in Table 1.

**Table 1:** Characteristics for the 196 men and 184 women from Sweden that participated in the study

	Men		Women	
	
	Diabetes		Diabetes	
	
	No (n = 184)	Yes (n = 12)	No (n = 174)	Yes (n = 10)
	
	Mean, median (5^th^, 95^th^%)	Mean, median (5^th^, 95^th^%)	Mean, median (5^th^, 95^th^%)	Mean, median (5^th^, 95^th^%)
Age (yr)				
Current	60, 59 (49, 75)	60, 60 (53, 67)	63, 61 (51, 77)	64, 64 (51, 74)
At diagnosis	-	52, 52 (30, 62)	-	55, 56 (45, 65)
Time since diagnosis (yr)	-	9, 5 (2, 26)	-	10, 10 (0, 27)
Body Mass Index (kg/m^2^)				
Current	28.6, 28.1 (23.5, 35.5)	29.4, 30.1 (22.4, 33.3)	27.9, 27.2 (21.3, 35.8)	30.4, 29.5 (26.6, 41.3)
At 25 years of age	23.4, 24.0 (20.0, 28.9)	26.0, 25.4 (21.3, 32.6)	22.0, 22.0 (17.9, 26.6)	23.0, 22.5 (19.7, 26.6)
Exposure (ng/g lipid)				
CB-153	430, 360 (110, 950)	670, 560 (360, 1600)	280, 240 (94, 620)	300, 230 (110, 810)
p,p'-DDE	800, 570 (110, 2100)	1100, 1100 (390, 2400)	800, 590 (100, 2300)	1600, 990 (300, 5300)

Out of the group of 813 men that responded to the questionnaire there were 617 subjects that did not participate in the clinical examination. The non-participants had similar age distribution (median 62 years, range 49–84) as the 196 participants (median 60, range 49–84). In addition, the BMI distributions were also very similar among the non-participants (median 26.5 kg/m^2^, range 17.1–39.9) and the participants (median 27.2, range 20.5–38.5). Out of the group of 779 women that responded to the questionnaire there were 595 subjects that did not participate in the clinical examination. There was neither any difference between the non-participating women and the 184 participants with respect to age (median 65 years, range 49–84 *versus *median 64 years, range 49–83) or BMI (median 26.5 kg/m^2^, range 17.1–39.9 *versus *median 26.2 kg/m^2^, range 19.7–38.2).

The study was performed in accordance with the Declaration of Helsinki and approved by The Lund University Ethic's Committee. All participants provided written informed consents.

### Blood sampling

Venous blood samples were drawn between 8.00 and 10.00 A.M, after 12 hr fasting, into sterile Vacutainer glass tubes (BD Vacutainer, Plymouth, UK). Serum was separated by centrifugation (4000 rpm, 10 minutes) and transferred to glass bottles and special tubes. All serum samples were stored at -80°C until analysis.

### Determination of CB-153 and p,p'-DDE in serum

The analyses were performed applying solid phase extraction using on-column degradation of the lipids and analysis by gas chromatography mass spectrometry as previously described [20-22]. Levels of detection, coefficients of variation and participation in quality control programs have been described in detail elsewhere [22].

### Determination of serum lipids by enzymatic methods

Serum concentrations of triglycerides and cholesterol were determined by enzymatic methods as described elsewhere [22]. The total lipid concentration in serum (g/L) was calculated by the following equations [23]:

Men: Total = 0.96 + 1.28*(triglycerides + cholesterol)

Women: Total = 1.13 + 1.31*(triglycerides + cholesterol).

### Statistics

The effect estimations (odds ratios, OR) between the exposure variables CB-153 and p,p'-DDE, respectively, and diabetes were obtained from logistic regressions. The exposure variables were treated as continuous variables. Due to the high correlation between CB-153 and p,p'-DDE (women r = 0.68; men r = 0.64) these variables were not included in the models simultaneously. As potential confounders we considered gender, current age (as continuous variable) and BMI at 25 years of age (as continuous variable). In addition, the exposure variables were categorized into three equally sized groups. For evaluation whether there were trends in the data with respect to prevalence of diabetes, Jonckheere-Terpstra test (StatXact Statistical Software) was applied. Moreover, separate analyses were performed for men and women. We did also test whether time elapsed since diagnosis of diabetes were correlated (Spearman's correlation test) with the exposure variables and age.

## Results

Twelve of the 196 men (6%) and 10 of the 184 women (5%) had diabetes. Five of the male diabetics had per oral antidiabetic drugs, two had a combination of per oral drugs and insulin, one had insulin only, and the remaining four were only on a diet. The corresponding figures for the female diabetics were four, two, one and three, respectively.

For the whole data set CB-153 was significantly associated with diabetes (an increase of 100 ng/g lipid corresponded to an OR of 1.16, 95% confidence interval [CI] 1.03, 1.32, p = 0.03). Among the men the corresponding OR was 1.20 (95% CI 1.04, 1.39, p = 0.01) and among the women 1.06 (95% CI 0.75, 1.50, p = 0.74). Regarding p,p'-DDE, there was for the whole data set a significant association between exposure and diabetes (an increase of 100 ng/g lipid corresponded to an OR of 1.05, 95% CI 1.01, 1.09, p = 0.006). The corresponding ORs were for the men 1.05 (95% CI 0.98, 1.11, p = 0.14) and for the women 1.05 (95% CI 1.01, 1.10, p = 0.02). None of the ORs changed more than marginally (<3%) by including the potential confounders. Moreover, excluding the two subjects with insulin only therapy changed the risk estimates <1%.

Using the exposure data categorized into tertiles there were for the whole data set significant positive trends between CB-153 and p,p'-DDE exposure, respectively, and diabetes (Table 2). Among the men significant positive trends between CB-153 exposure and diabetes (p = 0.005) and between p,p'-DDE exposure and diabetes (p = 0.04) were observed. Among the women the pattern was very similar regarding p,p'-DDE exposure and diabetes (p = 0.07), whereas no such association was observed for the CB-153 exposure.

**Table 2:** Prevalence of diabetes in relation to tertiles of CB-153 and p,p'-DDE in serum.

Gender	Diabetes	p for trend ^a^
	
Exposure (ng/g lipid)	Yes/No	
Male		
CB-153		
-290	0/64	
>290–475	4/61	0.005
>475	8/58	
p,p'-DDE		
-410	1/63	
>410–850	4/61	0.04
>850	7/60	
Female		
CB-153		
-180	3/57	
>180–290	4/57	0.94
>290	3/60	
p,p'-DDE		
-375	1/59	
>375–860	3/59	0.07
>860	6/56	

^a ^Jonckheere-Terpstra's test

Time elapsed since diagnosis of diabetes was among the men negatively correlated with CB-153 exposure (r_s _= -0.59, p = 0.04), whereas it tended to be positively correlated among the women (r_s _= 0.53, p = 0.12).

## Discussion

The main finding of the present study was that diabetics had significantly higher serum levels of both CB-153 and p,p'-DDE than non-diabetic control subjects, using both continuous and categorized exposure data. Gender stratified analyses showed among men consistent positive associations with CB-153, but a more ambiguous pattern with respect to DDE. In contrast, among the women the associations with p,p'-DDE were stronger than with CB-153. We have no biological explanation for this gender difference, which might be a random finding considering the relatively small size of the study.

Our overall results are in concordance with a number of previous epidemiological studies showing associations between T2DM and dioxin exposure [7-10], but also with PCB [11] and DDT/DDE [12] exposures. The epidemiological findings have some biological plausibility as TCDD in experimental studies of guinea pigs, mice and rats decreases cellular glucose uptake [4]. Moreover, it has recently been hypothesized that dioxins and dioxin-like PCBs could promote T2DM by interaction with peroxisome proliferators-activated receptor-γ, a ligand-activated transcription factor controlling lipid metabolism and homeostasis that is linked with T2DM [5,24]. There are no experimental data supporting that di-ortho PCB congeners such as CB-153, will have a diabetogenic effect by themselves, but CB-153 serves as a good proxy marker also for TCDD TEQ and the total POP derived TEQ.

An obvious caveat interpreting the epidemiological cross-sectional studies is to know the direction of the causality between POP exposure and T2DM. A reversed causality cannot be excluded, meaning that the disease affects the serum levels of POP. T2DM is associated with a variety of metabolic changes, which quite conceivably could alter the metabolism of POPs. T2DM can alter the pharmacokinetics of some drugs due to e.g. glycosylation of plasma proteins or displacement by increased plasma levels of free fatty acids, or through deteriorated kidney function [25] and also the activity of cytochrome P450 [26]. T2DM is also known to cause a dysregulation of fat metabolism, which in turn might influence the distribution and elimination of lipophilic compounds such as PCBs and dioxins [11]. If diabetics have a slower rate of excretion of TCDD and other POPs, this could account for the observed associations with T2DM [4]. The possibility of a slower elimination of dioxins in T2DM was, however, not supported by a recent study on Vietnam veterans, in whom no difference in TCDD half-life was found between diabetic and non-diabetic patients [27]. Moreover, if T2DM would slow down the excretion of POPs from the body, time elapsed since diagnosis of T2DM should be expected to be positively correlated with CB-153 in serum. Such a correlation was indicated, however not significant, among the women in the present study. On the other hand among the men there was a significant negative correlation between time since T2DM diagnosis and CB-153 in serum, which speaks against that the T2DM would have slowed down their POP excretion.

In the present study we used self-reported diabetes, and had no access to medical records. However, considering the age distribution, time elapsed since diagnosis and that only one man and one woman had insulin as single therapy, we feel convinced that almost all of the patients had a T2DM. Moreover, the prevalence figures in the present study (6% for men and 5% for men), are well in concordance with was observed in a recent Swedish population based study on similar age groups (about 7% in men and about 5% in women) [28]. Moreover, a sensitivity analysis excluding the two diabetic subjects with insulin only therapy showed that the risk estimates were changed with <1%.

When calculating BMI, we may have slightly underestimated the height at 25 years of age by measuring the current height, but we think that this has only introduced a minor non-differential misclassification.

## Conclusion

This cross-sectional study provides support for the hypothesis that POP exposure might contribute to type 2 diabetes mellitus. Even if we cannot exclude the possibility of a reversed causality, the presently observed negative correlation between time period elapsed since diabetes diagnosis and CB-153 level in serum, speaks for the hypothesis of POP as a risk factor.

## List of Abbreviations

BMI – Body Mass Index

CB-153 – 2,2',4,4',5,5'-hexachlorobiphenyl

p,p'-DDE – 1,1-dichloro-2,2-bis(p-chlorophenyl)-ethylene

OR – Odds Ratio

POPs – Persistent Organochlorine Pollutants

TEQ – Toxic Equivalent

T2DM – Type 2 diabetes mellitus

TCDD – 2,3,7,8-tetrachlorodibenzo-*p*-dioxin

## Competing interests

The author(s) declare that they have no competeting interests.

## Authors' contributions

LH initiated the project. LR and ARH performed the statistical analyses. All authors participated in the design of the study and of writing the manuscript. All authors have read and approved the final manuscript.
